# Loss of Ing3 Expression Results in Growth Retardation and Embryonic Death

**DOI:** 10.3390/cancers12010080

**Published:** 2019-12-29

**Authors:** Dieter Fink, Tienyin Yau, Arash Nabbi, Bettina Wagner, Christine Wagner, Shiting Misaki Hu, Viktor Lang, Stephan Handschuh, Karl Riabowol, Thomas Rülicke

**Affiliations:** 1 Institute of Laboratory Animal Science, University of Veterinary Medicine Vienna, 1210 Vienna, Austria; mail@tienyinyau.com (T.Y.); bettina.wagner@vetmeduni.ac.at (B.W.); s.m.hu@outlook.com (S.M.H.); viktor.b.lang@gmail.com (V.L.); thomas.ruelicke@vetmeduni.ac.at (T.R.); 2 Departments of Biochemistry & Molecular Biology and Oncology, Cumming School of Medicine, University of Calgary, Calgary, AB T2N 4N1, Canada; nabbi.arash@gmail.com (A.N.); karl@ucalgary.ca (K.R.); 3 Division of Immunology, Allergy and Infectious Diseases (DIAID), Department of Dermatology, Medical University of Vienna, 1090 Vienna, Austria; christine.wagner@meduniwien.ac.at; 4 VetImaging, VetCore Facility for Research, University of Veterinary Medicine Vienna, 1210 Vienna, Austria; stephan.handschuh@vetmeduni.ac.at

**Keywords:** Ing3, ING Proteins, insertional mutation, growth retardation, embryonic lethal

## Abstract

The ING3 candidate tumour suppressor belongs to a family of histone modifying proteins involved in regulating cell proliferation, senescence, apoptosis, chromatin remodeling, and DNA repair. It is a stoichiometric member of the minimal NuA4 histone acetyl transferase (HAT) complex consisting of EAF6, EPC1, ING3, and TIP60. This complex is responsible for the transcription of an essential cascade of genes involved in embryonic development and in tumour suppression. ING3 has been linked to head and neck and hepatocellular cancers, although its status as a tumour suppressor has not been well established. Recent studies suggest a pro-metastasis role in prostate cancer progression. Here, we describe a transgenic mouse strain with insertional mutation of an UbC-mCherry expression cassette into the endogenous *Ing3* locus, resulting in the disruption of ING3 protein expression. Homozygous mutants are embryonically lethal, display growth retardation, and severe developmental disorders. At embryonic day (E) 10.5, the last time point viable homozygous embryos were found, they were approximately half the size of heterozygous mice that develop normally. µCT analysis revealed a developmental defect in neural tube closure, resulting in the failure of formation of closed primary brain vesicles in homozygous mid-gestation embryos. This is consistent with high ING3 expression levels in the embryonic brains of heterozygous and wild type mice and its lack in homozygous mutant embryos that show a lack of ectodermal differentiation. Our data provide direct evidence that ING3 is an essential factor for normal embryonic development and that it plays a fundamental role in prenatal brain formation.

## 1. Introduction

The Inhibitor of Growth, member 3 (ING3), belongs to a family of proteins involved in regulating cell proliferation, senescence, apoptosis, chromatin remodeling, and DNA-repair. The family members are stoichiometric components of different histone acetyl transferase (HAT) and histone deacetylase (HDAC) complexes and function as regulators of cell growth through altering gene transcription as well as their ability to modify TP53 and NFKB1 activity [1]. ING3 is part of the NuA4/TIP60 HAT complex, specifically of the minimal NuA4 HAT complex consisting of EAF6, EPC1, ING3, and TIP60. Loss of TIP60 protein expression in mice results in embryonic death [2,3], whereas disruption of *Epc1* is controversial, resulting in a viable or embryonic lethal phenotype under homozygous conditions depending on the deleted or disrupted exons [4,5]. EAF6 has been attributed to a critical function in genomic regulation [6,7], yet no mutant mouse model has been described. 

The tumour suppressor roles of ING1 and ING2 have been well established whereas candidate tumour suppressor status remains for ING3, ING4, and ING5. Mice lacking ING1 or ING2 develop spontaneous tumours including lymphomas [8,9,10] and soft tissue sarcomas, respectively [11]. Furthermore, male mice lacking ING2 are infertile suggesting an important role in chromatin modification during germ cell development [11]. Mice deficient for ING4 are hypersensitive to lipopolysaccharide (LPS) treatment and display elevated cytokine responses, but fail to form spontaneous tumours [12]. Nevertheless, ING4 is downregulated or lost in multiple cancers and malignancies [13]. For the candidate tumour suppressor genes ING3 and ING5, no mutant mouse models have been described so far to support or weaken their tumour suppressor gene status and to further investigate their function in vivo.

*ING3* was initially discovered performing a computational search and further analysis showed that it modulated TP53 mediated transcription, cell cycle control, and apoptosis [14]. Furthermore, ING3 protein expression profiling in normal human tissues revealed high expression in cells undergoing rapid proliferation and renewal, which suggested a possible role in cellular growth and self-renewal [15]. It is frequently downregulated in human head and neck squamous cell carcinomas [16,17]. Loss of heterozygosity (LOH) of the *ING3* locus was also found in ameloblastoma [18], hepatocellular carcinoma [19,20], and colorectal cancer [21], supporting its tumour suppressor candidate gene status.

In contrast to its putative role as a tumour suppressor, a correlation study showed that strong nuclear staining for ING3 was associated with significantly worse five-year disease-survival compared with negative-to-moderate nuclear ING3 staining in malignant melanoma [22]. Furthermore, downregulation of ING3 in prostate cancer cell lines resulted in reduced growth along with upregulation in a cohort of prostate cancer tissues suggesting an oncogenic role in progression of prostate cancer metastases [23].

During experimental use of a previously generated fluorescence reporter mouse with ubiquitous expression of mCherry [24], we noticed an absence of homozygous transgenic offspring. With the exception of red fluorescence emission, hemizygous transgenic mice of this line are without any phenotypic changes and toxicity of mCherry was excluded as a reason for the absence of homozygous mutants. The mouse line was generated by lentiviral transgenesis and because the integration of viral vectors do not induce substantial sequence alterations at the integration site [25], the missing homozygous genotype among the offspring raised the suspicion of insertion of the viral vector into a vital endogenous gene. Within this work, we identified and characterized an insertional *Ing3* mutant mouse line that shows reduced embryonic growth and results in prenatal death of homozygous mutant embryos lacking ING3 expression. No differences between heterozygous mutants and wild type mice were detected. Due to the embryonic lethal phenotype of homozygous embryos, the tumour suppressor status of ING3 during the postnatal period could not be strengthened or weakened. Nevertheless, the findings suggest an important role of ING3 in embryonic development. 

## 2. Materials and Methods

### 2.1. Animal Husbandry, Breeding, Embryo Production, and Quantitation of Fluorescence

Animals were kept specific pathogen free according to FELASA [26] recommendations under controlled environmental conditions (temperature 22 °C ± 1 °C, relative humidity of 40–60%) in a facility for laboratory rodents. Food (regular mouse diet, ssniff Spezialitäten GmbH, Soest, Germany) and water were provided ad libitum. Mice were maintained in small groups in individually ventilated cages (Type IIL, Tecniplast, Buguggiate, Italy) lined with bedding material (Lignocel^®^, heat treated, Rettenmaier KG, Vienna, Austria) and enriched with nesting material (Pur-Zellin 4 × 5 cm; Paul Hartmann GmbH, Wiener Neudorf, Austria).

Experimental procedures were discussed and approved by the institutional ethics and animal welfare committee in accordance with good scientific practice guidelines and national legislation (animal breeding license number: BMWFW-68.205/0049-WF/V/3b/2015 and animal experiment license number: BMWF-68.205/0023-II/3b/2014 and BMWFW-68.205/0087-WF/V/3b/2015).

The *Ing3* insertional mutation was detected by chance in a breeding colony of the UbC-mCherry transgenic line B6(Cg)-*Tyr^c-2J^* Tg(UBC-mCherry)1Phbs/J, described as phenotypic inconspicuous at hemizygous conditions previously [24] (Jackson lab, Stock No. 017614). Due to the identification of the mutated endogenous gene, we suggest a renaming of the line according to the MGI Guidelines for Nomenclature for insertional mutations as B6(Cg)-Tyrc-2J Ing3^Tg(UBC-mCherry)1Phbs/J^. A second red fluorescence reporter mouse line, which was used as a control for quantification of mCherry expression, designated as C57BL/6NCrl-Tg(CAG-mCherry)690Biat, was also described previously [27]. For reconstitution of the *Ing3* mutation, an Ing3-P2A-eGFP expressing transgenic mouse line, designated C57BL/6N-TgTn(sb-CAG-Ing3-P2A-eGFP)774.1Biat was produced by transposon technology [28] and subsequent Cre mediated excision of a stop cassette (described in detail in Fink et al., manuscript in preparation).

For harvesting embryonic day (E) 3.5 and E10.5 embryos, UbC-mCherry transgenic mice were mated in the afternoon and female mice were checked for vaginal plugs the next morning and separated from the stud male. For reconstitution of ING3 expression, we bred hemizygous UbC-mCherry mutants with CAG-Ing3-P2A-eGFP transgenic mice and double transgenic offspring were backcrossed to UbC-mCherry mutants.

Quantification of overall fluorescence of UbC-mCherry and CAG-mCherry transgenic mice (five to seven weeks of age) was performed on a Xenogen IVIS 50 (PerkinElmer, Waltham, MA, USA) (fluorescence readout: “efficiency”) according to [27]. Potential double transgenic neonatal UbC-mCherry / CAG-Ing3-P2A-eGFP offspring were imaged on an IVIS Lumina S5 (PerkinElmer) (field of view 10, binning 16, exposure time 500 ms, fluorescence readout: “radiant efficiency”). “Efficiency” and “radiant efficiency” are instrument specific and illustrate relative fluorescence levels.

All animal numbers are indicated in the figure or figure legends.

### 2.2. Identification of Integration Locus of UbC-mCherry Transgenic Mice

The integration site of the transgene in UbC-mCherry transgenic mice was identified using linker mediated PCR (LM-PCR) [29]. Briefly, genomic DNA of UbC-mCherry mice was digested with MseI and resulting fragments were ligated to the linker oligos LMPCR-Linker (+): 5′-GTAATACGACTCACTATAGGGCTCCGCTTAAGGGAC-3′ and LMPCR-Linker (−): 5′-TAGTCCCTTAAGCGGAG-3′-NH_2_. A first round of PCR was performed using the primers FUW-*3LTR-Out-For* (5′-AGTGCTTCAAGTAGTGTGTGCC-3′) and *Linker-Primer* (5′-GTAATACGACTCACTATAGGGC-3′). The product of this amplification was then subjected to a nested PCR using the primers FUW-*3LTR-Inn-For* (5′-GTCTGTTGTGTGACTCTGGTAAC-3′) and *Nested-Primer* (5′-AGGGCTCCGCTTAAGGGAC-3′). PCR products were purified by gel extraction and sequenced using the FUW-*3LTR-Inn-For* primer. Sequences were blasted using the UCSC genome browser (http://genome.ucsc.edu) to determine integration sites. 

The UbC-mCherry transgenic line was generated by lentiviral mediated transgenesis, and we found that the primers FUW_3LTR-For-Out and FUW_3LTR-For-Inn primers are located within the 3′ LTR, and therefore also present in the 5′ LTR after integration of the lentiviral vector. Thus, a new primer FUW_3For-Out for confirmation of the integration sites was designed upstream of the 3′ LTR for this purpose. Additionally, genomic primers upstream (mC-L1_5Gen-For-Out, 5′-ACAAAAGCCTGCCAGATCATATA-3′) and downstream (mC-L1_3Gen-Rev-Out, 5′-CTCACGGTGGACACTTGG-3′) of the insertion site of the *Ing3* locus were designed to amplify a 351 bp wild type fragment. The primer pair mC-L1_5Gen-For-Out and FUW_5LTR-Rev-Out (5′-CTCTCGCACCCATCTCTCTC-3′) amplifies an 859 bp fragment at the 5′ junction, whereas the primer pair FUW_3-For-Out (5′-GGTGGGTTTTCCAGTCACAC-3′) and mC-L1_3Gen-Rev-Out amplifies a 764 bp transgenic fragment at the 3′ junction. PCR reactions were performed according to the manufacturer’s recommendation.

PCR cycling conditions were as follows: initial denaturation at 95 °C for 2 min followed by 35 cycles of 95 °C for 15 s, 62 °C for 30 s, and 72 °C for 2 min, and a final extension at 72 °C for 7 min.

### 2.3. PCR Genotyping of Pre-Implantation Stage Embryos

For genotyping of preimplantation stages, blastocysts were flushed at E3.5 from uterus horns of superovulated females. Harvested blastocysts were washed once in PBS and separately placed in microtubes with homogenization buffer. Further preparation and analysis was performed according to the protocol 3 of tissue lysates for PCR [30]. The primers and PCR conditions were the same as described below for UbC-mCherry genotyping.

### 2.4. PCR Genotyping of Ear Biopsies and Amnion of E10.5 Embryos

Genomic DNA from ear biopsies and amnion was extracted using the Tris-NaCl-EDTA-SDS (TNES) extraction protocol. Briefly, the tissue was incubated in 100 µL TNES buffer (10 mM Tris-Cl, 400 mM NaCl, 100 mM EDTA, 0.6% SDS) supplemented with proteinase K (98 µL buffer + 2 µL proteinase K (Thermo Fisher Scientific GmbH, Dreieich, Germany; cat# EO0491)). It was then incubated at 55 °C overnight, then 35 µL of salt solution (5 M NaCl) were added, vortexed, and centrifuged at 13,000 rpm on a table top centrifuge. The supernatant (100 µL) was transferred to a new tube, 100 µL ice cold 100% ethanol were added, and centrifuged at 13,000 rpm to precipitate the DNA. The supernatant was removed and the precipitate was washed with 500 µL 70% ethanol and centrifuged again at 13,000 rpm. The pellet was dissolved in 50–100 µL of Tris-EDTA buffer (10 mM Tris-Cl, 1 mM EDTA, pH 8.0).

A sample of 0.5 µL of DNA was subjected for PCR reactions in a total volume of 15 µL per reaction according to the OneTaq Quick-Load protocol (New England Biolabs GmbH, Frankfurt am Main, Germany; cat# M0486L).

For UbC-mCherry genotyping, a three primer PCR (mC-L1_5Gen-For: 5′-AGGCTGTCAGTCTACTCCCTCT-3′, mC-L1_3Gen-Rev: 5′-CACAGGTGAGGAGCAAAGTCTC-3′, FUW-5LTR-Rev: 5′-AGAGAGCTCCTCTGGTTTCCCT-3′) will amplify a 306 bp wild type and/or a 631 bp transgenic fragment. PCR conditions were as follows: initial denaturation at 95 °C for 2 min followed by 35 cycles of 95 °C for 30 s, 60 °C for 30 s, and 68 °C for 30 s, and a final extension at 68 °C for 5 min.

For CAG-Ing3-P2A-eGFP genotyping, the primers LSL-CAG-For (5′-GCCTCTGCTAAACCATGTTCATGC-3′) and LSL-ING3-Rev (5′-GCATTCTGCACCTGCAGATCC-3′) will amplify a 337 bp transgenic fragment. PCR conditions were as follows: initial denaturation at 95 °C for 2 min followed by 35 cycles of 95 °C for 30 s, 62 °C for 30 s, and 68 °C for 30 s, and a final extension at 68 °C for 5 min.

### 2.5. Western Blot Analysis

E10.5 embryos were harvested and snap frozen in liquid nitrogen and lysed in Laemmli buffer without bromphenol blue and β-mercaptoethanol. The samples were diluted tenfold for protein concentration determination using the BCA protein assay kit according to the manufacturer’s protocol (Pierce^TM^ BCA Protein Assay Kit, Thermo Fisher Scientific GmbH, Dreieich, Germany; cat# 23227). Afterwards, bromphenol blue and β-mercaptoethanol were added to the samples, heated to 95 °C, and 20 µg of total protein were loaded and separated on a 12% SDS polyacrylamide gel. It was then transferred onto Immobilon-P PVDF membranes (Merck KGaA, Darmstadt, Germany; cat# IPVH00010) by semidry electro-blotting (Hoefer, Inc., Holliston, MA, USA;). After blocking for 2 h with 5% skim milk (Bio-Rad Laboratories Ges.m.b.H., Vienna, Austria; cat# 1706404) in PBS with 0.05% Tween-20 (PBST) (Sigma-Aldrich Handels GmbH, Vienna, Austria; cat# P9416), the membranes were cut and incubated with primary goat anti-ING3 antibody, dilution 1:800 (Abcam, Cambridge, UK; cat# ab115470), and mouse anti-α-tubulin antibody, with a dilution of 1:5000 (Merck KGaA, Darmstadt, Germany; cat# CP06) in 5% skim milk in PBST overnight at 4 °C. Membranes were washed twice with PBST and incubated with HRP-conjugated secondary rabbit anti-goat antibody, dilution 1:10,000 (Bio-Rad, cat# 1721034) or goat anti-mouse antibody, with a dilution of 1:40,000 (Bio-Rad, cat# 1706516) in 5% skim milk in PBST for 1 h at room temperature and washed three times with PBST. Protein signal was visualized using the ECL detection system (Pierce^TM^, cat# 32209) according to the manufacturer’s instructions. A B16 mouse melanoma cell line (ATCC, LGC Standards GmbH, Wesel, Germany; cat# CRL-6322) extract was used as control and processed the same way as the embryos. Densitometric readings were performed using Adobe Photoshop. The whole western blot with molecular weight marker is shown in Figure S1.

### 2.6. Histological Analysis

E10.5 embryos were harvested, placed in 10% neutral buffered formalin (VWR, cat# 9713.1000), incubated at 4 °C overnight, and subsequently transferred to 70% ethanol. Tissues were paraffin embedded and sectioned (6 µm) and slides were stored at −80 °C until staining.

For heat induced epitope retrieval (HIER) Epitope Retrieval Solution 1 (citrate based pH6; Leica, cat# AR9961) was used on the Leica Bond Rx system (Leica, Concord, ON, Canada) at 95 °C for 10 min. Slides were put into the autostainer, treated with Peroxidase block, followed by 30 min incubation at room temperature with anti-ING3 antibody clone 2A2 (1:100 dilution) [15] in Signal Stain antibody diluent (Cell Signaling, Danvers, MA, USA; cat# 8112L). Expression of ING3 was visualized by incubation for 30 min with LabeledPolymer-HRP anti-mouse (Dako, Agilent Technologies Canada Inc., Mississauga, ON, Canada; cat# K4000) reagent and followed by a 10 min incubation with DAB+ substrate and chromogen (Dako, cat#K3468). Slides were counterstained with Mayer’s hematoxylin (Dako, cat# S3309). 

### 2.7. µCT analysis

Embryos were harvested at E10.5, fixed in formalin over night at 4 °C, and subsequently stored in 70% ethanol. Embryos were stained with 0.1% phosphomolybdic acid hydrate (Sigma-Aldrich, cat# 79560-100G) in 70% ethanol for six hours on a horizontal shaker. Subsequently, specimens were washed in 70% ethanol and mounted in heat-sealed plastic pipette tips in 70% ethanol. In total, four wild type, four hemizygous, and three homozygous embryos were subject to µCT analysis. µCT scans were acquired with an XRadia MicroXCT-400 (Carl Zeiss X-ray Microscopy, Pleasanton, CA, USA) at 40 kVp source voltage and 200 µA current using the 4X detector assembly. Projection images (1014*1014 pixel, camera binning = 2) were recorded with an exposure time of 4 s per projection and an angular increment of 0.2 ° between projections. Tomographic sections were exported as DICOM sequences. Isotropic voxel size in the reconstructed image volumes was 3.56 µm. For visualization, image volumes were imported into Amira 6.5. (FEI SAS, Mérignac, France (part of Thermo Scientific™)). 

## 3. Results

**Figure 1:**
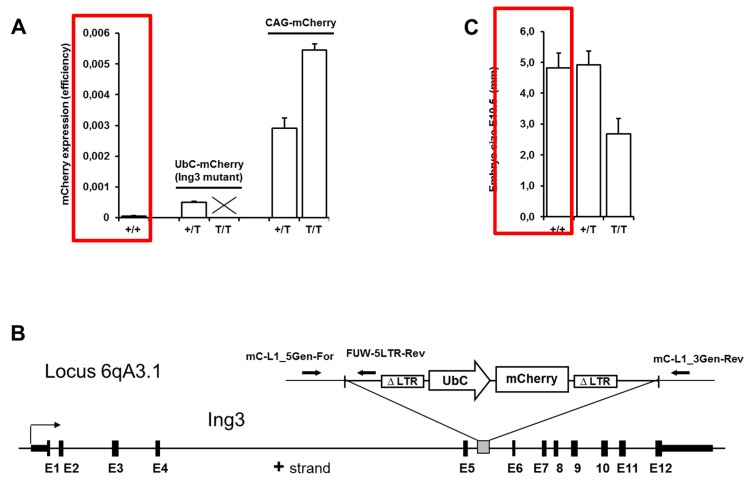
Quantification of overall fluorescence, schematic view of UbC-mCherry integration site, and embryo size. (**A**) Overall fluorescence of wild type (+/+; *n* = 4) and hemizygous (+/T; *n* = 8) UbC-mCherry mice, and hemizygous (+/T; *n* = 4) and homozygous (T/T; *n* = 4) CAG-mCherry mice. (**B**) Integration site of the UbC-mCherry cassette at 6qA3.1 disrupting the *Ing3* locus. (**C**) Embryo size of wild type (+/+; *n* = 9), heterozygous (+/T; *n* = 19), and homozygous (T/T; *n* = 8) *Ing3* insertional mutants at E10.5.

After breeding and application of a previously established novel non-invasive genotyping procedure for fluorescently labelled transgenic mice [27], we noticed the absence of viable homozygous UbC-mCherry transgenic mice (first described in [24]) in the offspring of hemizygous × hemizygous matings (see Figure 1A and Table 1). To eliminate any possibility of mCherry toxicity at high expression levels, we compared the overall expression levels (fluorescence efficiency) to the viable CAG-mCherry hemizygous and homozygous transgenic mice (first described in [27]) that is approximately 5-fold and 9-fold higher, respectively, compared to hemizygous UbC-mCherry transgenic mice (Figure 1A). 

It has been reported that lentiviral vector-mediated transgenesis frequently leads to integrations in the middle of transcribed regions and thereby induces insertional mutations in endogenous genes [31]. The integration site of the UbC-mCherry cassette was detected at 6qA3.1 disrupting the *Ing3* locus by unknown mechanisms (Figure 1B). Possible explanations for silencing of an endogenous gene by insertion of a transgene into an intron are transcriptional interferences or transcription stop by retroviral poly(A) signal.

Mapping the integration site of the UbC-mCherry cassette to the locus of the tumour suppressor candidate ING3 prompted us to further investigate the homozygous lethal phenotype. To analyze the impact of the insertional mutation on early development of homozygous embryos, genotyping of blastocysts obtained from hemizygous × hemizygous breeding revealed the presence of homozygous viable blastocysts with the expected frequency (Table 1). The latest time point during pregnancy at which viable homozygous embryos were detected was at E10.5. These embryos were approximately half the size of hemizygous and wild type littermates (Figure 1C).

**Figure 2:**
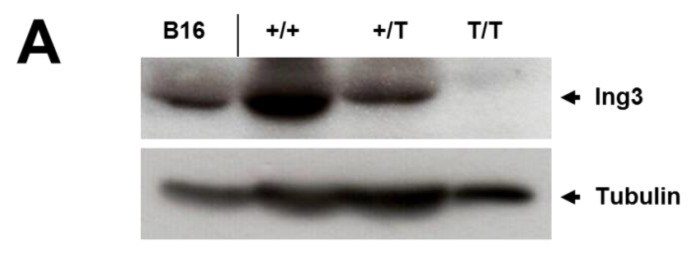

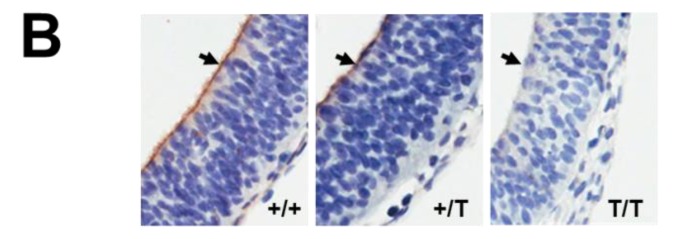
Protein expression in whole E10.5 embryos and the developing brain of E10.5 embryos. (**A**) Western blot analysis for ING3 of wild type (+/+; *n* = 1), heterozygous (+/T; *n* = 1), and homozygous (T/T; *n* = 2) whole embryos and intensity ratio readings ING3/Tubulin. (**B**) Immunohistochemisty for ING3 of wild type (+/+; *n* = 2), heterozygous (+/T; *n* = 2), and homozygous (T/T; *n* = 1) embryos. A representative section of the developing brain is shown and indicated by arrows. Magnification 40×.

Western blot analysis and immunohistochemistry confirmed the absence of ING3 protein in homozygous UbC-mCherry embryos at E10.5 (Figure 2A, B), confirming disruption of the ING3 locus. Furthermore, immunohistochemistry revealed strong ING3 expression in the fetal brain of developing wild type and heterozygous embryos at E10.5 (Figure 2B).

**Figure 3:**
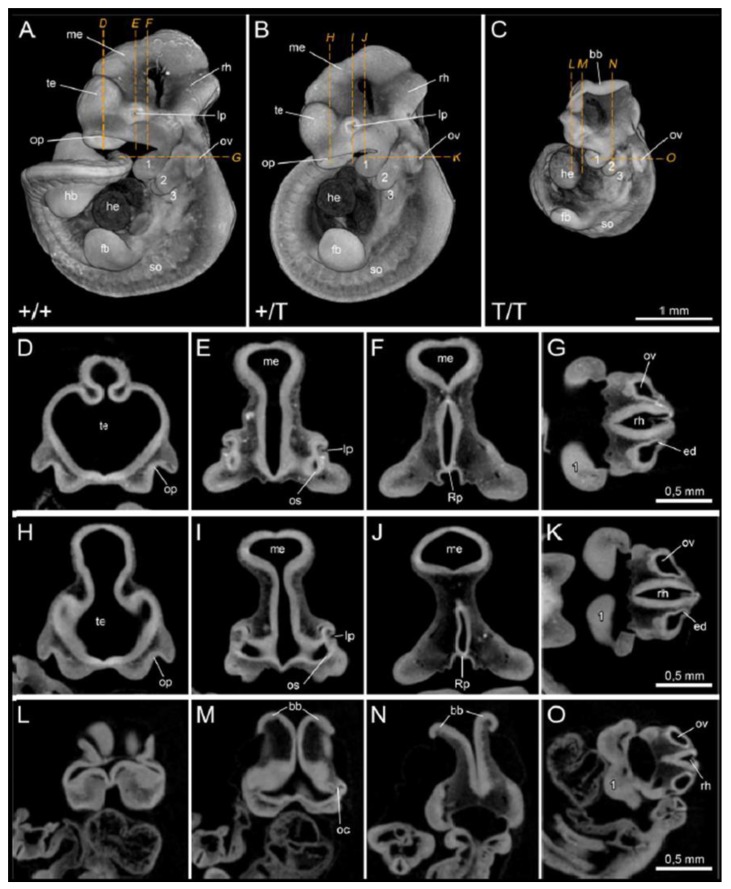
µCT analysis of E10.5 embryos. Volume renderings show lateral view of (**A**) wild type (+/+; *n* = 4), (**B**) heterozygous (+/T; *n* = 4), and (**C**) homozygous (T/T; *n* = 3) embryos. Virtual µCT sections of (**D**,**E**,**F**,**G**) wild type, (**H**,**I**,**J**,**K**) heterozygous, and (**L**,**M**,**N**,**O**) homozygous embryos. Note that in (**A**) the tail bud was clipped unintentionally during specimen preparation and in (**B**) the hindlimb bud and tail bud are located behind the embryo head and thus are hidden in the image. (1, 2, 3: branchial bars 1–3; bb: brain bulges; ed: endolymphatic duct; fb: forelimb bud; hb: hindlimb bud; he: heart; lp: lens pit; me: mesencephalon; oc: optical cup; op: olfactory pit; os: optic stalk; ov: otic vesicle; rh: rhombencephalon; Rp: Rathke’s pouch; rs: retinal sheet; te: telencephalon).

To further investigate the anatomical developmental defects, µCT analysis of E10.5 embryos was performed. For morphological evaluation of wildtype, heterozygous, and homozygous embryos, we referred to the atlas of embryonic development of the house mouse [32]. Analyzed E10.5 wild type embryos showed typical Theiler stage 17 characteristics. Externally, branchial bars 1–3 are visible, the forelimb buds (fb) are elongated, the hindlimb buds (hb) are bulged, and the tail bud is well developed (Figure 3A, Video S1). 30–35 somites were counted, which is slightly less than described for Theiler stage 17. Differences in somite count might be attributed to different specimen contrast in light microscopic and µCT evaluation, as during this stage the most cranial somites become more and more indistinct. In the head, the three primary brain vesicles already started to develop into the five secondary brain vesicles, which is obvious in the two distinctive telencephalon hemispheres of wild type and heterozygous embryos (Videos S1 and S2). In wild type embryos, the lens anlage (lens pit, lp) shows a deep indentation (Figure 3A,E), which is accompanied by a thickened retinal sheet (rs) and a well-developed optical stalk (os) (Figure 3E). The olfactory pits (op) are well visible (Figure 3A,D). In the developing mouth, the opening of Rathkes pouch (Rp) is not yet constricted (Figure 3F). The otic vesicle (ov) is closed, pear-shaped, and the anlage of the endolymphatic duct (ed) can be distinguished (Figure 3G). No differences between E10.5 wild type embryos and heterozygous embryos were found (Figure 3B,H–K, Video S2). 

In addition to the fact that homozygous mutant embryos are much smaller than their littermates as already shown in Figure 1C, they display severe developmental defects. Externally, branchial bars 1–3 are visible, the forelimb buds (fb) are less elongated, and 29 somites were counted (Figure 3C, Video S3). The hindlimb buds are just visible as a thickened ridge, and the tail bud is present in homozygous embryos (Video S3). Slight differences in defects can also be observed between individual embryos and are due to the technical setup of the timed mating. The developmental range at E10.5 depends on the actual fertilization time point and may vary up to several hours between pregnant females if the female and stud male are paired overnight.

Significant differences of the homozygous embryos compared to hemizygous and wild type embryos were observed in the head region and central nervous system. The brain was not closed, and instead the folding brain bulges (bb) were widely opened at the anterior neuropore (Figure 3C, L–N, Video S3). The optical cups (oc) can be seen, yet no indentation of the lens anlage (lens pit, lp) appears (Figure 3M). Olfactory pits (op) are absent (Figure 3C,L), the otic vesicles (ov) are closed and round (Figure 3O), and Rathkes pouch (Rp) is not visible (Figure 3N). The heart (he) is approximately the same size in wild type, heterozygous, and homozygous animals (Figure 3A–C).

**Figure 4:**
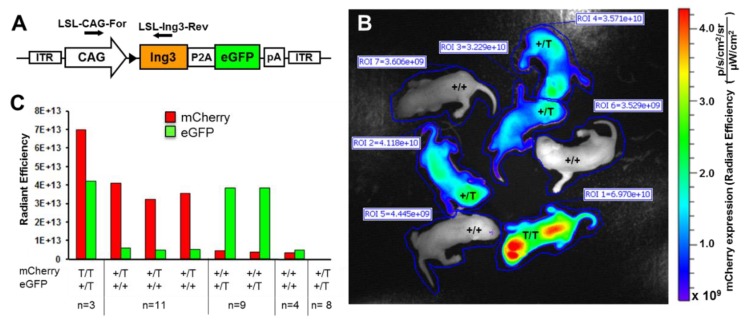
Reconstitution of ING3 by ubiquitous expression of Ing3-P2A-eGFP. (**A**) Schematic view of the *Ing3* transposon construct. (**B**) In vivo imaging showing mCherry expression of different genotypes in a representative litter of a UbC-mCherry, CAG-Ing3-P2A-eGFP double mutant bred to a hemizygous UbC-mCherry mouse. (**C**) mCherry and eGFP expression with corresponding genotypes of UbC-mCherry and CAG-Ing3-P2A-eGFP transgenic pups (homozygous (T/T), hemizyous (+/T) and wild type (+/+)). Total numbers of animals per genotype are indicated in the figure. Position of genotyping primers are indicated by arrows.

The mutant ING3 mouse model was identified as an insertional mutation and lack of ING3 expression was confirmed by western blot analysis and immunohistochemistry. Nevertheless, to provide a final proof that ING3 is the only and definitive cause of this serious phenotype, we reintroduced ING3 by ubiquitous expression. We therefore bred the insertional mutant UbC-mCherry line to CAG-Ing3-P2A-eGFP transgenic line (schematic view of the vector construct is shown in Figure 4A) and after backcrossing the hemizygous double mutants to hemizygous UbC-mCherry mice we obtained for the first time homozygous viable UbC-mCherry transgenic mice with physiologically normal appearance, suggesting successful reconstitution of the insertional *Ing3* mutation. Homozygous UbC-mCherry animals were easily detected by in vivo imaging in neonates showing increased fluorescence levels (almost 2-fold) compared to hemizygous neonates (Figure 4B, C). To facilitate the in vivo imaging procedure, the ING3 in CAG-Ing3-P2A-eGFP transgenic mice is linked to eGFP by P2A [33] and homozygous mutant mice also display green fluorescence (Figure 4B), suggesting the expression of Ing3-P2A-eGFP from the transgenic construct.

## 4. Discussion 

The results clearly show the developmental failure upon loss of ING3 expression. µCT analysis revealed a general delay in growth in homozygous embryos with major growth restriction in the closing of the brain buds and further formation of the three primary brain vesicles (prosencephalon, mesencephalon, and rhombencephalon) seen in heterozygous and wild type embryos at E10.5 [34]. The brain is closed at Theiler stage 14 (E9.0, 13–20 somites) in normal development before limb buds start to appear [32]. 

Neurulation starts with the folding of the neural tube around E6.5 from the ectoderm. Remarkably, the most severe developmental malformations in homozygous mutants were found in structures developing from the ectoderm, namely the delayed closure of the posterior neuropore and failure of the closure of the anterior neuropore that is still widely open at E10.5. Also, we observed no formation of the lens pit (lp), the olfactory pits (op), and the delayed development of the otic vesicles (ov). Thus, we hypothesize that ING3 is an essential factor during ectoderm differentiation. However, since homozygous embryos die after E10.5, we cannot exclude the possibility that other defects in tissues from a different origin are also affected at a later developmental stage.

ING3 activates the TP53 responsive promoter p21^WAF1^ and BAX, negatively controls cell growth, and increases apoptosis in a TP53 dependent manner in colon cancer cells [14]. Furthermore, overexpression of ING3 in gastric cancer cells resulted in apoptosis with increased BAX and CASPASE-3 expression and down-regulated expression of the anti-apoptotic protein BCL2. ING3 was also shown to drastically inhibit the levels of p-PI3K and p-AKT that reduce cell proliferation [35]. PTEN, a tumour suppressor gene that is embryonic lethal in knockout mice, counteracts the PIP3 phosphorylation of p-PI3K and the proliferative effect of PI3K/AKT signaling by dephosphorylating PIP3 to PIP2. Pten deficient embryos appear delayed at E7.5 with ectoderm development and morphologically distinct mesoderm, but no headfold was visible. The embryo in general appeared compact and less organized with severe overgrowths in the cephalic region in E8.5 embryos, leading to death at E9.5 [36]. The involvement of ING proteins in cell cycle control and down regulation in of INGs and in particular ING3 in multiple cancers review in [13] suggests that loss of ING3 can result in increased proliferation and/or reduced apoptosis in vitro and supports its status as a putative candidate tumour suppressor. However, our findings in vitro and in vivo have suggested ING3 is expressed in rapidly growing cells [15,23] and our current study shows that loss of ING3 reduces growth in homozygous mutant embryos while no gross phenotype was seen in heterozygous embryos, suggesting that lower ING3 levels (Figure 2A) are sufficient for normal development. Reduced growth was also shown for homozygous ING1 knockout mice with targeted deletion of the critical exon 2 (null for all isoforms) [8] and in a gene trap knockout mouse with deletion of the long isoform ING1b [9]. Mouse embryonic fibroblasts (MEFs) isolated from Ing1b^-/-^ mice showed increased proliferation that is further increased in ING1b^-/-^;Trp53^-/-^ double knockout MEFs compared to wild type MEFs, which indicates a Trp53 independent mechanism. This increased proliferation in ING1b null MEFs is counterintuitive to the reduced body weight of ING1b null mice. In contrast to our ING3 mutant, the ING1 null mutant showed a slightly reduced number of homozygous mice born after heterozygous crosses as the expected Mendelian distribution [8]. Negative control of the cell cycle after overexpression of ING1 or ING2 in U2OS [37] or normal skin fibroblast [38] cells is consistent with the observations of overexpression of ING3 in cancer cells [14,35] given their opposite involvements in HDAC and HAT complexes, respectively. p33^ING1^ (ING1b) and p33^ING2^ (first identified as ING1L) share approximately 60% of sequence homology, and one may substitute for another in some assays [39]. This may also be the case for ING4 and ING5, as the phylogenetic analysis of the ING family suggests [40]. Due to the location of ING1 and ING2 on the same chromosome in the mouse (chromosome 8), double mutants are hardly obtained by standard breeding and may require the production of a new double knockout mouse. This double mutant may then display a similar homozygous embryonic lethal phenotype, as seen in the ING3 mutant mouse.

In summary, loss of ING1 protein expression shows reduced growth in homozygous live born mice that might be amplified in ING1 and ING2 double mutants, whereas ING3 deficiency shows reduced growth in embryos that leads to abortion latest at E11.5. This is caused by reduced proliferation, increased apoptosis, or senescence related to the DNA damage response and including involvement of ING3 in PI3K/AKT signaling [41,42]. 

To further investigate the role of ING3, we recently generated a conditional knockout mouse model that will allow for detailed investigations of ING3 function during pre- and postnatal development utilizing tissue and developmental stage specific Cre mouse lines. Additionally, the EUCOMM cell line used for generation of the tm1a allele (knockout first) contains an artificial exon that is spliced in frame with the N-terminal sequence of ING3, expressing the LacZ reporter. This will simplify the expression analysis according to protein levels of ING3 in embryos, neonates, and in adult mice by LacZ staining, and thus will not be dependent on antibody specificity. 

Considering the tumour suppressor candidate status of ING3, we have not seen any abnormal developments in ING3 mutant heterozygous animals compared to wild type mice as reported previously [24]. Nevertheless, a conditional knockout mouse, together with the constitutive *Ing3* insertional mutant and the conditional Ing3 transgenic mouse will provide valuable tools for further analyzing the functions of ING3 in in vivo cancer models.

## 5. Conclusions

We here show that loss of ING3 expression in an insertional mutant mouse model of *Ing3*, a candidate tumour suppressor of the ING family, leads to severe growth restriction and embryonic death latest at E11.5. Since most developmental defects were detected in tissues derived from the ectoderm, we hypothesize that ING3 is a major factor for ectoderm differentiation.

## Figures and Tables

**Table 1:** Genotypes of Ubc-mCherry born mice, female of male ratio, and genotype of blastocysts from hemizygous × hemizygous matings.

Genotype	+/+	+/T	T/T
Number of born mice	113(36%)	199(64%)	-
Female/Males	56/57	102/97	-
Number of blastocysts	10(23%)	24(56%)	9(21%)

100% refer to the total number born mice and total number of blastocysts.

## Supplementary Materials

The following are available online at https://www.mdpi.com/2072-6694/12/1/80/s1, Figure S1: Western blot raw data, Video S1: µCT video of rotating wild type embryo, Video S2: µCT video of rotating heterozygous embryo, Video S3: µCT video of rotating homozygous embryo.
