# Supplementary material for: Loss of Ing3 Expression Results in Growth Retardation and Embryonic Death
Source: Cancers (Basel). 2019 Dec 29;12(1):80. doi: 10.3390/cancers12010080 (PMC7017303; doi:10.3390/cancers12010080)
Supplement: Supplementary file 1 [file cancers-12-00080-s001.pdf]

Supplementary Materials: Loss of Ing3 Expression Results in Growth Retardation and Embryonic Death

Dieter Fink, Tien Yin Yau, Arash Nabbi, Bettina Wagner, Christine Wagner, ShiTing Misaki Hu, Viktor Lang, Stephan Handschuh, Karl Riabowol and Thomas Rülicke

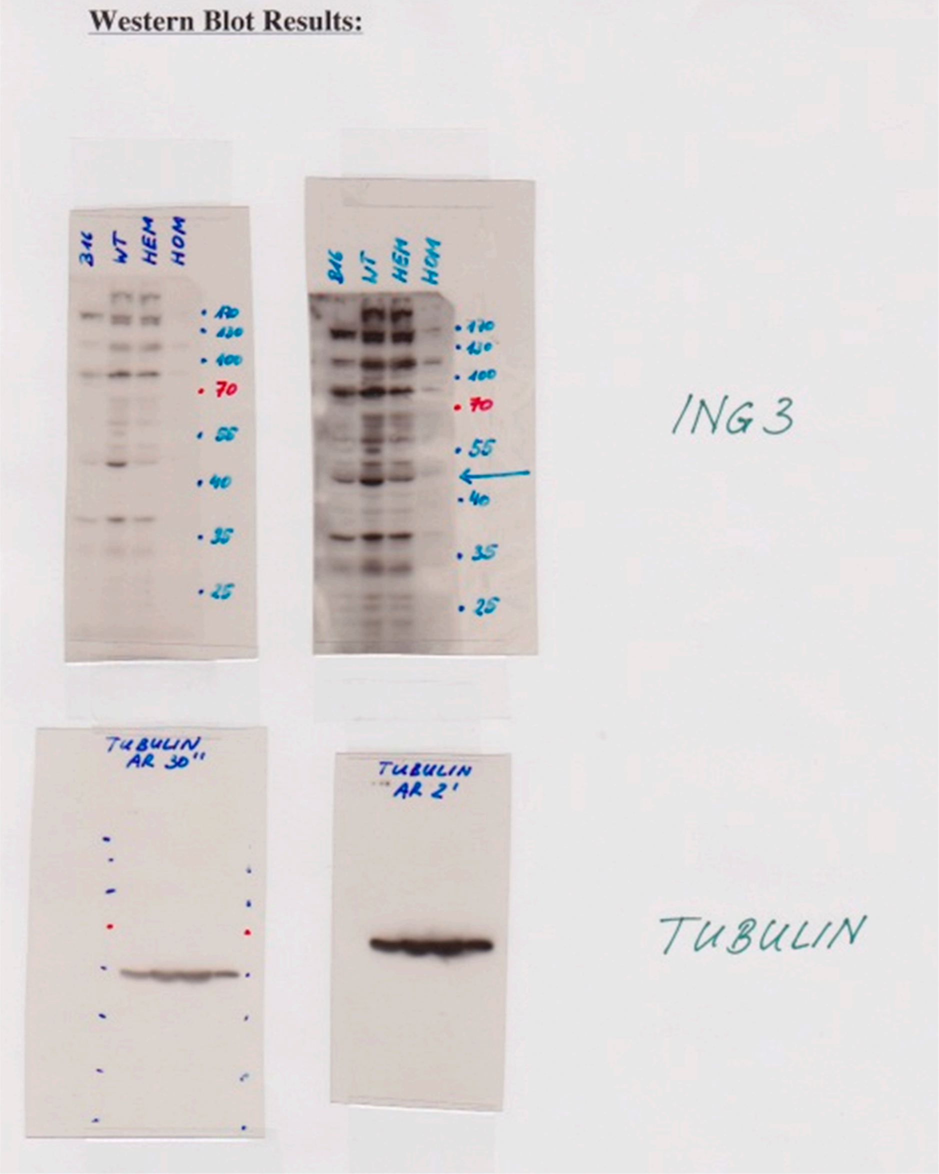

Figure S1. Western blot raw data. Two exposure times of the western blot (left panel 30 s, right panel 2 min) for Ing3 (top) and tubulin loading control (bottom) were conducted. Molecular weight marker (kD) is labelled on the Ing3 blot while only indicated with dots (red dot for 70 kD) for the tubulin blot. Loading from left to right: B16, wild type (WT), hemizygous (HEM), homozygous (HOM). The expected molecular weight for Ing3 is 47 kD. Reduced protein levels are clearly assessable for animals with the half gene dosage (HEM, indicated by blue arrow, between 40 and 55 kD) compared to WT and not detectable in the HOM lane. We participated in the Abreview program (www.abcam.com) and rated the antibody used for the western blot with two stars out of five due to multiple bands, indicating its low specificity.

© 2019 by the authors. Licensee MDPI, Basel, Switzerland. This article is an open access article distributed under the terms and conditions of the Creative Commons Attribution (CC BY) license (http://creativecommons.org/licenses/by/4.0/).
